# Book Review

**Published:** 1903-09

**Authors:** 


**Our Baby.** By Mrs. J. LANGTON HEWER. Eighth Edition. Pp. viii., 154. Bristol: John Wright & Co. 1902.—The fact that this little book has reached the eighth edition in a comparatively short time shows that it supplies a want, and is appreciated by the public. The directions as to the general management of the baby are excellent, but we cannot help thinking that the chapter on "baby's illnesses" is a mistake, and that the book would have been more valuable to the general reader without it.

**Annual Report of the Smithsonian Institution for the year ending June 30th, 1901.**—Washington: Government Printing Office. 1902.—This volume, the first printed in the twentieth century, commences with a history of the foundation and subsequent progress of the Institution. We are proud to find that the Smithsonian Institution owes its being to an enlightened Englishman, James Smithson, at one time a vice-president of the Royal Society, who died in Genoa, in 1829, leaving his entire estate to the United States of America, "to found at Washington, under the name of the Smithsonian Institution, an establishment for the increase of and diffusion of knowledge among men." After ten years of debate Congress accepted the trust, and created by enactment, an establishment called by the name of the Smithsonian Institution, to be governed by a board of Regents responsible to Congress. The first meeting of the Regents took place in 1846. Its first work consisted, in the main, of the publication of original memoirs, and their free distribution to important libraries throughout the world; giving popular lectures, stimulating scientific work by providing apparatus, and by making money grants to worthy investigators, etc. Gradually out of the collections which had been kept in the Patent Office, the private collection of Smithson, etc., there grew up a Smithsonian Museum, which has since developed into the United States National Museum, which is now "the foremost collection in the world in everything that relates to the natural history, ethnology, geology, paleontology of that portion of North America now the United States." In the course of years a large library of 150,000 volumes has been formed, the major portion of which has been deposited in the Library of Congress. Other work which has been entrusted to the Institution is the Bureau of American Ethnology, the Astrophysical Observatory, and the National Zoological Park. "The establishment of the latter was intended primarily to preserve the vanishing races of mammals on the North American Continent; but it has also assumed the general features of a zoological park, affording the naturalist the opportunity to study the habits of animals at close range." Of the value of this park to students of natural history we have testimony in the interesting paper, in the latter part of the volume, by E. T. Seton, on "A Study of Animals in relation to their Natural Environment." Of the catholicity of the aims of the Institution we have ample proof, when we find that a "Children's Room" has lately been provided in the Smithsonian Building, in which has been arranged a natural history exhibition "for the especial benefit of children, little children, who did not care for long, hard names, and who could not see objects on high shelves." The rest of the volume is taken up as usual with a valuable collection of monographs by the best-known scientific experts in the world. These include papers by Lord Kelvin, on "Ether and Gravitational Matter Through Space"; on "Bodies Smaller than Atoms," by J. J. Thompson; "Wireless Telegraphy," by G. Marconi, etc. Popular tastes are also catered for, as there is an account of Santos Dumont circling the Eiffel Tower in his air-ship, and also an account of the Paris to Berlin automobile race. As is always the case in these reports, the volume is profusely and beautifully illustrated.

**Medico-Chirurgical Transactions.** Vol. LXXXV. London: Longmans, Green, & Co. 1902.—One of the most noteworthy papers in this volume is that on malignant endocarditis, by Drs. Poynton and Paine. They show that there is a group of cases of malignant endocarditis which is rheumatic in nature, and due to a diplococcus rheumaticus; that a diplococcus isolated from certain cases of malignant endocarditis in man will produce, not only malignant endocarditis in rabbits, but a condition indistinguishable from the disease they believe to be rheumatic fever; and, further, that by these diplococci every grade of valvulitis, from simple to malignant and from malignant to simple, can be produced. These researches appear to establish two most important points—the first of which is, that rheumatic fever must be an organismal disease due to a specific diplococcus; and the second is, that this same rheumatic microbe is capable of producing in rabbits either simple rheumatic fever or malignant endocarditis, or both.

**Transactions of the Association of American Physicians.** Vol. XVII. Philadelphia. 1902.—Of the many noteworthy papers here printed, one on pneumococcic arthritis is of especial interest: the author, Dr. Herrick, gives an epitome of the literature of this condition, and sums up its clinical characteristics; he points out that exploratory aspiration is the only means of ascertaining the nature of the joint lesion, and that incision and drainage are usually necessary. In connection with pneumonia, a paper by Dr. William Travis Howard, jun., gives an interesting review of the pathology of labial and nasal herpes, and of herpes of the body occurring in acute croupous pneumonia, and their relation to the so-called herpes zoster.

**Transactions of the American Dermatological Association, 1901.** New York: Rooney & Otten Printing Co. 1902.—The statistics of a large number of cases are always interesting; and 22,455, although rather less than last year, give very fair data. Sixteen cases of "acanthus nigricans" are reported from New York, 171 dermatitis epidemica (Savill), two pemphigus vegetans, two pityriasis rubra (relatively small), and four of urticaria pigmentosa. There is nothing special to remark about the presidential address, by Dr. F. J. Shepherd (Montreal). During a discussion which followed the exhibition of twenty-six lantern slides, showing the clinical features and histo-pathology of small-pox, by Dr. S. Politzer, of New York, several members related cases of the unvaccinated disease which were of a mild type, and which tend to make the diagnosis of the complaint more complicated than ever. Thirty-six pages are devoted to some very interesting papers and discussions on "Diseases of the Nails." Cases were related of leucopathia nigricans, which were associated with alopecia areata, both which seemed to have had their starting point in neurotic depression. Some interesting evidence was produced, showing that psoriasis of the nails was relatively more frequent than eczema of the same; but, of course, eczema (general or local) is much more frequent than psoriasis, in the ratio of about 10 to 1. Dr. Montgomery (San Francisco) traces the streaking of the skin in "nævus linearis" to developments of the fœtus when the embryonic layers are still a plastic mass. Dr. W. A. Pusey (Chicago) extols the use of the X-rays in the treatment of many additional skin affections. The *Transactions* are well got up, and are very carefully indexed.

**The Commemoration Volume of the Epidemiological Society, containing an Account of the Foundation of the Society and an Index of the Papers read at its Meetings.** 1902.—Along with the *Transactions of the Epidemiological Society for 1902*, we have received the above volume, which gives a most interesting account of the foundation of the Society and of the valuable work it has done for the community at large during the last half-century. Perhaps the most celebrated work achieved was the report of the committee "On the state of Small-pox in England and Wales and other Countries" presented to Parliament, and ordered to be printed by the House of Commons in 1855, which is described by the writer of the history as "almost epoch-making." Reading this little book, one cannot but be struck most forcibly with the enormous difference fifty years has made in our knowledge of sanitary science.

**Index-Catalogue of the Library of the Surgeon-General's Office, United States Army.** Second Series. Vol. VII., Hernia—Inquiry. Washington: Government Printing Office. 1902. Pp. 1,003.—The present volume of this invaluable and monumental work contains 6,225 author-titles, 13,179 subject-titles of separate books and pamphlets, and 32,522 titles of articles in periodicals. It is absolutely indispensable to all medical libraries.

**The Journal of Tuberculosis.** Edited by KARL VON RUCK and SILVIO VON RUCK. Vol. V. No. 1. January, 1903. Asheville, N.C.: A. H. McQuilkin.—This is a quarterly magazine devoted to the prevention and treatment of tuberculosis. The relations between human and bovine tuberculosis are the subject of a paper by Professor Baumgarten, of Tübingen, and the address of Dr. Robert Koch, delivered at the international conference in Berlin, on "The Transmission of Bovine Tuberculosis to Man," is reproduced. Other abstracts on this subject are here found, and an editorial article gives a judicious summary of the evidence now obtainable. The editor is a well-known and enthusiastic student of everything relating to tubercle, and we are glad to include this journal in our exchange list.

**Medical Annual, 1903.** Bristol: John Wright & Co.—The twenty-first volume of this useful annual does not differ notably from its predecessors, nor does the list of its contributors. A general summary of the year's work by Dr. Hobart Amory Hare commences the "Dictionary of Materia Medica and Therapeutics" (Part I.), and the "Dictionary of Treatment." Part II. similarly commences with a general review of medical and surgical progress for 1902. Of the many useful lists contained in the book, that of sanatoria for the treatment of tuberculosis will be much in demand. X-rays, high-frequency currents and light treatment are much in evidence throughout the volume, and much is expected from these agents at present: we read that "too often, in the past, electrical treatment has suffered from over-enthusiasm, and it would be a matter for serious regret were anything to occur which would bring on the inevitable reaction in thought."

**The Nurse in Hot Climates.** By EDWARD HENDERSON M.D. Pp. 47. London: The Scientific Press Ltd. 1903.—This little work of 47 pages has been revised and reprinted from *The Hospital*. Any nurse who contemplates a new experience in a hot climate would be wise to study its pages closely, as it contains much instruction and good common sense. The difference in the details of nursing in London and Calcutta must be considerable, but any English trained nurse would soon master these differences by the aid of this guide.

**Tropical Diseases.** By PATRICK MANSON, C.M.G., M.D. New and Revised [Third] Edition. Pp. xxiv., 756. London: Cassell and Company, Limited. 1903.—We are not surprised to see the issue of a third edition of this most excellent manual. In these days of rapid advance in all branches of medical science the publication of each new edition of a text-book means an increase in its size, and although there has necessarily been an addition of seventy pages to this edition, the book still keeps to its handy form, and as a concise and practical guide to medical men practising in tropical countries its merits are undoubted, and our former opinions of the work are fully maintained. The subject of malaria is gone into very fully, and we notice the addition of many useful plates to illustrate the anatomy and development of the mosquito which will enable observers who have the means at their command to distinguish the various forms of the insect that are now of such interest to the pathologist. The author acknowledges the good work done by the schools of tropical medicine, and gives an account of the recent experiments of American and other observers as to the part played by a species of mosquito in the spread of yellow fever. These researches explain, in Dr. Manson's opinion, many facts as tó infectivity and period of incubation which were before obscure. The chapters on animal parasites and their associated diseases are especially good and instructive—the description of the life history and best methods of demonstrating their presence in the blood or tissues will be of great value to practitioners. Particular stress is laid on the possibility of the anæmia due to ankylostomiasis being overlooked or attributed to other causes—a practical point which should be kept in mind. In connection with this subject it is also shown that the embryo of A. Duodenale may gain admission into the system through the skin, a fact observed first as the result of an accident, and subsequently verified by experiment. Of filariasis the author gives a very complete account. It is a subject full of interest. He recognises four different species of the parasite as infecting man, and in a careful summing up gives his reasons for regarding elephantiasis as a filarial disease. In our opinion the book is a most valuable one, and we can only repeat what we expressed in a former review of it, that it should be in the possession of all medical men whose practice lies in tropical countries. A very complete index adds much to its value.

**Muco-membranous Colitis.** By Dr. FROUSSARD. Pp. 15. London: Baillière, Tindall and Cox. 1903.—A short essay on this intractable malady concludes with the statement that "very often this disease is greatly improved by a water cure. In France, Plombières and Chatel-guyon are the two places where it is treated with the greatest success. Plombières is especially recommended for the cases which are very painful and accompanied by aggravated intestinal cramps and other nervous symptoms." We can but welcome any suggestion which gives a chance of cure in a chronic case of this most unsatisfactory disease.

**Transactions of the College of Physicians of Philadelphia.** Vol. XXIV. Philadelphia: William J. Dornan. 1902.—A notable feature of this volume is its series of obituary notices of men who are worthy to be remembered. That of Dr. J. M. DaCosta, the author of the well-known book on *Medical Diagnosis*, is written by Dr. J. C. Wilson; that of Dr. John Ashhurst, jun., by Dr. Richard H. Harte, who concludes as follows:—"To know John Ashhurst was to respect him; to secure the privilege of his friendship was to love and revere him with a love and reverence seldom given to mortals to obtain." The notice of Dr. Alfred Stillé is written by Dr. William Osler, who remarks "that the physician of the last century, whose 'floruit' came in the fourth, fifth, and sixth decades, is of all men the most to be envied. Coming out of the wilderness in which we had wandered for two thousand years, he entered the promised land, and under the leadership of Laennec and Louis, of Skoda and of Virchow, he saw the heathen dispossessed and the profession at last enter upon a heritage of scientific medicine. Of such was Dr. Stillé, who, born in 1815, began his life work with the generation which saw the new pathology and the new clinical methods. Dr. George C. Harlan writes of Dr. William Fisher Norris that "he filled a conspicuous position in the medical profession of Philadelphia faithfully and well, and will long be remembered and honoured in its history."

**The Treatment of Fractures.** By CHARLES LOCKE SCUDDER, M.D. Third Edition. Pp. 485. London: W. B. Saunders & Company. 1902.—This is a useful book. The most striking feature about it is its wealth of illustrations in connection both with the diagnosis and treatment of fractures. They are very clear, and the majority are of much practical value. A few skiagrams are reproduced, and there are many pictures made from skiagrams, but they are, as the preface states, "the combined interpretation of the plate made by skilled observers who were in every instance familiar with the clinical aspect of the case." This looks as if they were somewhat imaginative drawings. We had rather expected in this new edition to find a chapter devoted to the question of early movement and massage in fractures, as it has been so prominently before the profession, in this country at any rate, but a chapter has been devoted to the ambulatory treatment of fractures. The author is not in favour of this method. An interesting account is also given of the use of the X-rays for diagnosis and the interpretation of skiagrams, and the views expressed by the American Surgical Association on the medico-legal aspect of X-ray work are given in full. It seems curious to English readers to find that all fractures of the neck of the femur are treated of together, irrespective of their position, and to read that whether the fracture is intra or extra-capsular is of little moment. The author has endeavoured to find a term which is a little more expressive of the condition met with in what are termed simple and compound fractures. The term open fracture, proposed as a substitute for compound, is a good enough one, but to call the other variety a "closed" fracture rather suggests that it has been closed by the surgeon. We must confess, however, that no really suitable term occurs to us. We are advised by the author not to operate for fractured patella without fully explaining the risks to the patient, and never unless we are skilled in antiseptic methods, and this is, of course, very sound advice. We look in vain for descriptions of the various methods of operating employed in this country. Mr. Barker's well-known plan is not even alluded to. We entirely agree with the author in not advising operation in the ordinary form of fracture-dislocation of the spine. The amount of writing in the book is perhaps not so great as one might imagine from its size, as the print is large, and the very numerous pictures take up much room. It is a thoroughly practical work.

**The Surgical Treatment of Ulcer of the Stomach.** By C. MANSELL MOULLIN, M.D. Pp. 53. London: John Bale, Sons & Danielsson, Ltd. 1902.—This collection of papers, which does not mean to be a treatise on gastric ulcer, will be found useful to the physician as well as to the operating surgeon, as it puts in a concise form the special symptoms and physical signs to be looked for in cases requiring operative treatment, and it also gives shortly the choice and the mode of operation required. We quite agree with the author when he says that "if only properly selected cases of ulcer of the stomach were treated surgically, before the condition of the patient has been allowed to become hopeless, many lives that are now sacrificed would be saved, and an enormous amount of pain and suffering would be prevented."

**Tumours, Innocent and Malignant.** By J. BLAND-SUTTON. Third Edition. Pp. xii., 556. Cassell & Company, Ltd. 1903.—The present edition of this extremely interesting and valuable book contains some important additions in the text and figures. The author has wisely dissociated the old "myeloid sarcoma" from the sarcomata, under the more appropriate name of "myeloma," and "deciduoma malignum" has been provisionally placed amongst the sarcomata. The term "epithelioma" has been replaced by that of "squamous-celled cancer"—a phrase which is pathologically more correct, though more alarming to the sufferers from this disease. Two of the most interesting chapters of the newer additions are those treating of renal and adrenal tumours and of uterine myo-fibromata. The evil effects of pregnancy and the dangers which may ensue after the menopause in patients affected with myo-fibromata are clearly set forth. The painstaking labours of the author have already become classical, and this third edition has brought them up to the knowledge of the present day. The classification is sound, the descriptions clear, and the illustrations are numerous, helpful, and well executed.

**Saunders' Year Book of Medicine and Surgery.** Under the general editorial charge of George Gould. *Surgery.* Pp. 671. London: W. B. Saunders & Company. 1903. Only one-half of the book is devoted to general surgery, the other portion being devoted to obstetrics, gynecology, orthopedic surgery, ophthalmology, diseases of the nose, throat and ear, and anatomy. The existence of such books is a necessity for those who find it impossible to wade through the voluminous literature which appears nowadays on all professional subjects. The author has been ably helped by well-known specialists in the various departments; and the present volume is worthy of its predecessors and one of the best of the numerous "annuals."

**Anleitung zur Diagnose und Therapie der Kehlkopf-, Nasen- und Ohrenkrankheiten. Vorlesungen gehalten in Fortbildungscursen für practische Aerzte.** Von Richard Kayser. Zweite vermehrte und verbesserte Auflage. Pp. vi., 178. Berlin: S. Karger. 1903. —The account given of the diagnosis and treatment of the affections of the nose, throat and ear in this little book is concise and practical. In 170 pages all the most important affections of these regions are described and the appropriate treatment for each outlined. We consider the book an ideal one of its kind. We have noticed no omissions of importance, and no insertions which could have been omitted with advantage. It would be an excellent thing if every medical student or general practitioner could be induced to read, mark, learn, and inwardly digest the contents of this or some such compendium dealing with this branch of the healing art. The book is not intended for specialists, but for those who have neither the time nor the opportunity for studying encyclopædic works on the subjects dealt with. We accordingly find special stress laid upon those methods of diagnosis and treatment which every practitioner should be prepared to carry out. That the book has been well received is shown by the fact that a second edition has been so soon called for. In this second edition we note that many of the newest methods of diagnosis and treatment are alluded to; for example, the use of Killian's specula, of Grey's solution of cocaine in aniline oil and alcohol, and of gelatine for the arrest of epistaxis. The book is, moreover, agreeably written and sufficiently illustrated.

